# Clinical Phenotyping and Biomarkers in Spinal and Bulbar Muscular Atrophy

**DOI:** 10.3389/fneur.2020.586610

**Published:** 2021-01-20

**Authors:** Elina Millere, Dmitrijs Rots, Ieva Glazere, Gita Taurina, Natalja Kurjane, Viktorija Priedite, Linda Gailite, Kaj Blennow, Henrik Zetterberg, Viktorija Kenina

**Affiliations:** ^1^Department of Neurology and Neurosurgery, Children's Clinical University Hospital, Riga, Latvia; ^2^Department of Doctoral Studies, Riga Stradins University, Riga, Latvia; ^3^Scientific Laboratory of Molecular Genetics, Riga Stradins University, Riga, Latvia; ^4^Department of Neurology, Pauls Stradins Clinical University Hospital, Riga, Latvia; ^5^Department of Biology and Microbiology, Riga Stradins University, Riga, Latvia; ^6^Department of Medical Genetics and Prenatal Diagnostics, Children's Clinical University Hospital, Riga, Latvia; ^7^Outpatient Service Centre, Pauls Stradins Clinical University Hospital, Riga, Latvia; ^8^BIOCON Medical Laboratory, LCC BIOCON, Riga, Latvia; ^9^Department of Psychiatry and Neurochemistry, Institute of Neuroscience and Physiology, the Sahlgrenska Academy at the University of Gothenburg, Mölndal, Sweden; ^10^Clinical Neurochemistry Laboratory, Sahlgrenska University Hospital, Mölndal, Sweden; ^11^Department of Neurodegenerative Disease, University College London Queen Square Institute of Neurology, London, United Kingdom; ^12^UK Dementia Research Institute at University College London, London, United Kingdom; ^13^Rare Disease Centre, Riga East Clinical University Hospital, Riga, Latvia

**Keywords:** Kennedy disease, spinal and bulbar muscular atrophy, phenotype, clinical features, biomarker, neurofilament

## Abstract

**Background:** Spinal and bulbar muscular atrophy (SBMA) or Kennedy disease [OMIM: 313200] is a rare X-linked neuromuscular disease. Patients commonly present with muscle cramps, tremors, leg weakness, dysarthria and dysphagia.

**Methods:** We deeply phenotyped and evaluated the possible extent of affected systems in all patients with SBMA in Latvia (*n* = 5). In addition, neurophysiological studies and blood analyses were used to perform a molecular diagnosis and evaluate biochemical values. We analyzed neurofilament light (NfL) as a possible biomarker.

**Results:** Neurological examination revealed typical SBMA clinical manifestations; all patients had small or large nerve fiber neuropathy. Three of five patients had increased neurofilament light levels.

**Conclusion:** The study confirms the systemic involvement in patients suffering from SBMA. Increased NfL concentration was associated with either peripheral neuropathy or decreased body mass index. The complex phenotype of the disease should be kept in mind, as it could help to diagnose patients with SBMA.

## Introduction

Spinal and bulbar muscular atrophy (SBMA), also known as Kennedy disease, is a rare X-linked neuromuscular disease (1). It is caused by a CAG repeat expansion resulting in >35 CAGs in the first exon of the androgen receptor (*AR*) gene (polyglutamine disease) (2). The mutant AR aggregates in the nucleus of a cell and results in direct toxicity to the cell (mostly, lower motor neurons and myofibrils), which clinically results in slowly progressive adult-onset lower motor neuron damage and primary myopathy early in the course of the disease (3, 4). Interestingly, myopathic changes in transgenic mouse models of SBMA were observed prior to any motor neuron degeneration, illustrating motor neuron involvement only in the later stages of SBMA (5–7). Additionally, the polyglutamine expansion causes loss of receptor activity and results in androgen insensitivity.

The reported prevalence of SBMA is highly variable. Recent data in Italy indicated a SBMA prevalence of 2.58/100,000 in the male population (8), while it is higher in regions with a suggested founder effect, such as in the Vasa region of Finland where the estimated prevalence is 15/100,000 male inhabitants (8, 9). The exact prevalence, however, is still unknown, because SBMA is thought to be underdiagnosed (10).

Patients with SBMA commonly present with slowly progressive muscle weakness as the main clinical feature, which typically starts in the lower limbs proximally and progresses during the course of the disease to also involve the bulbar and distal limb muscle groups (2, 3). Other symptoms such as cramps, myalgia and postural hand tremors are frequently reported long before the onset of muscle weakness. Lower motor neuron signs are persistent, including diminished or absent deep tendon reflexes and fasciculations in the limb muscles, tongue, and chin, as well as muscle atrophy (2–4, 11). The onset of the neurological symptoms is variable and ranges from 20 to 60 years, but SBMA typically manifests during the fourth or fifth decade of life. The CAG repeat size inversely correlates with the disease onset, with longer repeats associated with earlier onset but not with disease progression or severity (4). Additionally, androgen insensitivity leads to gynecomastia, reduced fertility, and erectile dysfunction (2, 12).

Recent studies and separate case series have found that SBMA might also affect other parts of the nervous system and can be associated with metabolic alterations. Additional neurological features include peripheral neuropathies and dysfunction of the autonomic system, while metabolic alterations include dysregulation of both glucose homeostasis (glucose intolerance and diabetes) and lipid metabolism (dyslipidaemia). These findings suggest that SBMA is a systemic disorder (2, 12).

Determining disease progression in clinical trials with limited time frame for a slowly progressing disease could be challenging. Several possible neuronal and muscle biomarkers of SBMA severity have been previously studied. Creatinine, instead of creatinine kinase, demonstrates capabilities as a biomarker by reflecting SBMA severity via muscle mass decrease (13, 14). Blood concentrations of the axonal damage marker neurofilament light (NfL) has been reported to be unchanged in patients with SBMA (14).

The involvement of various systems has been previously assessed separately, analyzing one or several systemic manifestations mostly in a limited number of patients with SBMA. Therefore, in this study, we deeply phenotyped all patients with SBMA in Latvia to evaluate the possible simultaneous extent of the involved systems previously reported or suggested to be affected in SBMA, as well as analyzed possible biomarkers of SBMA.

## Materials and Methods

### Study Participants

All patients diagnosed with spinal and bulbar muscular atrophy in Latvia (five unrelated Caucasian male patients) were included in this study. For the comparison of plasma NfL levels, 21 healthy male controls without known neurological disease or neurologic symptoms were enrolled in this study as a control group.

### Clinical and Laboratory Examination

Clinical characteristics, including neurological status testing, 6-min walking testing, and hand grip testing, were evaluated by a certified neurologist at the time of enrolment into the study.

All patients underwent neurophysiological studies, including nerve conduction study (NCS), needle electromyography (EMG), and quantitative sensory testing (QST). For analysis of autonomic nervous system (ANS) function, heart rate variability (ANS Analysis Professional) and sympathetic skin response testing were performed. 2 days before the ANS tests, the patients stopped medication(s) that could influence the results.

Blood samples were used for investigating the following biochemical and hormonal profiles: creatinine, creatine phosphokinase, lipidogram, thyroid hormones (thyroid stimulating hormone and free thyroxine), testosterone, and glycated hemoglobin.

The NfL concentration in plasma was determined using the single molecule array (Simoa) NfL assay (Quanterix, Billerica, MA). For a quality control (QC) sample with a concentration of 15.8 pg/mL, repeatability was 5.2% and intermediate precision was 5.2%. For a QC sample with a concentration of 50 pg/mL, repeatability was 2.7% and intermediate precision was 3.2%. The lower limit of quantification was 1.9 pg/mL. The samples were analyzed by board-certified laboratory technicians who were blinded to the clinical data. The levels with controls were compared using Mann-Whitney *U*-test.

### Molecular Analysis

The CAG repeat analysis in the *AR* gene was performed as previously described (15). All analyses were performed using capillary electrophoresis, and the results were confirmed by Sanger sequencing for two patients with the shortest CAG repeats using the BigDye Terminator Kit v.3.1 (Thermo Fisher Scientific, USA), according to adapted manufacturer's protocol. The number of CAG repeats for longer alleles were calculated based on fragment size.

### Ethics

The study was approved by the Central Medical Ethics Committee of the Republic of Latvia (Nr.3/18-03-21). All individuals signed an informed consent form and allowed anonymised publication of their clinical information and photos.

## Results

The study included all patients with SBMA in Latvia (*n* = 5) that were 34–68 years old. Patient characteristics are shown in Table 1. The CAG repeat numbers were variable (range 47–54). All patients had no positive family history for SBMA or other neuromuscular diseases. Two of the patients also had autoimmune thyroiditis, and one of the patients had primary arterial hypertension. No other comorbidities were reported during the study. For two of the patients, SBMA manifested during their thirties, although the earliest onset was at the age of six based on the patient's anamnesis data (the first complains was inability to perform the same sports activities as other children). Almost all patients indicated their initial symptoms as general fatigue and muscle cramps.

**Table 1:** Patient characteristics.

	**Patient 1**	**Patient 2**	**Patient 3**	**Patient 4**	**Patient 5**
Age at examination / age at onset^*^ (years)	38/33	46/36	35/14	34/6	68/45
CAG repeats	50	48	54	47	53
Family history	Negative	Negative	Negative	Negative	Negative
Children	1 daughter	1 son	-	-	2 sons
Comorbidities	-	Autoimmune thyroiditis	Autoimmune thyroiditis	-	Primary Arterial Hypertension
Body Mass Index (kg/m^2^)	30.4	25.4	22.5	18.7	24.6

* *Self-reported by patient*.

**Figure 1:**
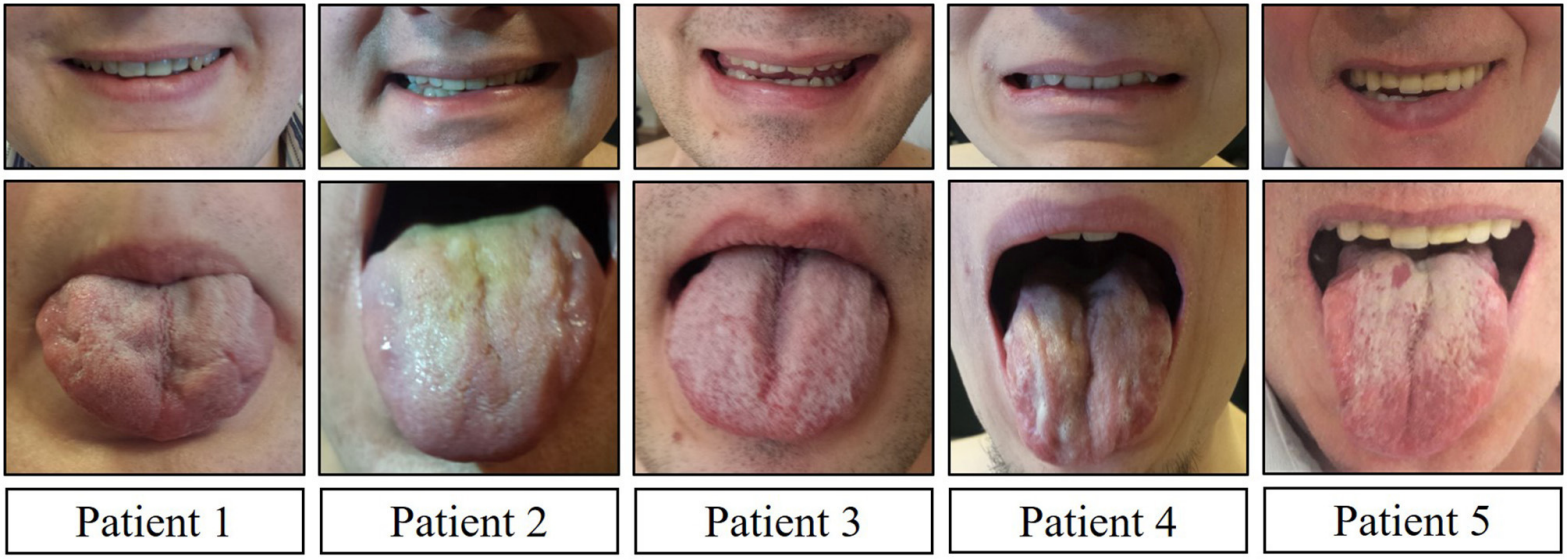
Facial muscle weakness and tongue atrophy.

Clinical and neurological examination revealed limb weakness in all patients. Weakness was more prominent proximally in the lower extremities and asymmetric between the left and right side for almost all patients (Table 2). Patient 4, with the earliest manifestation of the disease, also had the most severe weakness in the extremities. Facial muscle weakness, diffuse fasciculations, absent tendon reflexes, and tongue atrophy were present in all subjects (Figure 1). Two patients had dysphagia and two had dysarthria. During examination, all patients had prominent hand tremor, and Patient 3 also presented with bilateral myotonia in the palms. Sensory assessment revealed glove and stocking-like sensory impairment as hyperesthesia for Patients 1 and 3 and hypoesthesia for Patient 5. Gynecomastia was observed in four out of five patients. Patients without gynecomastia had low body mass index (BMI), 18.7 kg/m^2^. All patients noted sweating disturbances, but due to the small sample size and subjectivity of the complaints, it was questionable whether these complaints could be associated with BMI. All patients had decreased 6-min walking distances (Table 3) (16). All patients had clearly decreased hand grip testing results with poor scores for both hands.

**Table 2:** Clinical features.

	**Patient 1**	**Patient 2**	**Patient 3**	**Patient 4**	**Patient 5**
Initial symptoms	General fatigue	General fatigue	General fatigue	General fatigue Muscle cramps Muscle pain	Fatigue and muscle pain in lower limbs
Gynecomastia	Asymmetrical, since 18 y.o.	Asymmetrical, since 38 y.o.	Asymmetrical, since 14 y.o.	-	Symmetrical, since 40 y.o.
Limb weakness	Asymetric tetraparesis	Asymetric tetraparesis	Asymetric tetraparesis	Asymetric tetraparesis	Asymetric tetraparesis
Other symptoms	Hand tremor since 30 y.o., increased sweating	Hand tremor since 30 y.o., increased sweating	Hand tremor since 15 y.o., myotonia in hands, increased sweating	Hand tremor since 25 y.o., decreased sweating	Hand tremor, decreased sweating
Dysphagia	-	+	-	-	+
Dysarthria	-	-	-	+	+
Dysphonia	-	-	-	-	+
Facial muscle weakness (Figure 1)	+	+	+	+	+
Tongue atrophy (Figure 1)	+	+	+	+	+
Tendon reflexes	Absent	Absent	Absent	Absent	Absent
Fasciculations	Diffuse	Diffuse	Diffuse	Diffuse	Diffuse
Muscle cramps				Diffuse	Diffuse
Tremor	+	+	+	+	+
Sensory assessment	Hyperesthesia (glove and stocking type)	N	Hyperesthesia (glove and stocking type)	N	Hypoesthesia (glove and stocking type)

**Table 3:** Patient functional characteristics.

	**Patient 1**	**Patient 2**	**Patient 3**	**Patient 4**	**Patient 5**
6-min walking distance in meters [norm value (16)]	540 (580)	510 (580)	500 (580)	345 (580)	195 (580)
Hand grip test right/left in kg [age specific norm (17)]	44/41 (47/47)	31/26 (47/45)	30/30 (47/47)	20/10 (47/47)	25/20 (40/38)

In all patients, needle EMG showed clear signs of neurogenic changes in all examined muscles, with large polyphasic motor unit potentials, fibrillation potentials, and fasciculations (Table 4). Motor and sensory nerve conduction velocities and compound muscle action potentials and sensory action potential amplitudes were abnormal for two out of five patients. Patient 1 had signs of sensory demyelinating polyneuropathy with decreased sensory conduction velocities, and Patient 5 had severe motor sensory demyelinating axonal polyneuropathy. QST revealed abnormalities of tactile thresholds and mechanical pain perception in all patients, which was isolated in C sensory fiber types in two patients, A-δ fiber types in one patient and abnormalities in both types of sensory fibers in two patients.

**Table 4:** Neurophysiological findings.

	**Patient 1**	**Patient 2**	**Patients 3**	**Patient 4**	**Patient 5**
Nerve conduction studies	Sensory demyelinating polyneuropathy	Normal	Normal	Normal	Severe motor, sensory demyelinating axonal polyneuropathy
Electromyography	Neurogenic changes	Neurogenic changes	Neurogenic changes	Neurogenic changes	Neurogenic changes
Quantitative sensory testing	A delta type fiber dysfunction	C type fiber dysfunction	C type fiber dysfunction	A delta and C type fiber dysfunction	A delta and C type fiber dysfunction
The heart rate variability (ANS Analysis Professional)	Normal	Cardial autonomic neuropathy (tachycardia)	Sympatotonuss (tachycardia)	Parasympatotonuss	Normal
Sympathetic skin response test	Normal	Increased latency, decreased amplitude	Increased latency	Increased latency, decreased amplitude	Normal

Evaluation of the ANS via heart rate variability (ANS Analysis Professional) showed marked differences between patients. Patients 1 and 5 had no abnormalities; however, three patients had signs of ANS dysfunction. Patient 2 had signs of cardiac autonomic neuropathy (tachycardia), Patient 3 had sympathetic dysfunction, and Patient 4 had parasympathetic dysfunction. Sympathetic skin response testing results indicated that three out of five patients had peripheral sympathetic nervous system dysfunction, with all of them (3/5) demonstrating increased latency, and Patients 2 and 4 also had decreased amplitude during examination.

All patients had markedly elevated creatine phosphokinase levels, with the highest result reaching 5,000 U/L. All patient serum creatinine levels were within the normal range. To determine possible metabolic dysfunction linked to the pathogenesis of the disease, lipidogram, thyroid hormones, testosterone, and glycated hemoglobin levels were analyzed (Table 5). Two patients had increased testosterone levels, indicating androgen resistance. All patients had dyslipidaemia. Regarding thyroid function, two patients had autoimmune thyroiditis according to antibody (anti-thyroid peroxidase) and thyroid ultrasonography findings. Only Patient 3 had a subclinical decrease in thyroid function with increased thyroid stimulating hormone, despite demonstrating free thyroxine levels within the normal range.

**Table 5:** Biochemical and hormonal profile.

	**Normal range**	**Patient 1**	**Patient 2**	**Patient 3**	**Patient 4**	**Patient 5**
Cholesterol (mmol/l)	<5.0	4.04	4.74	5.19	4.83	4.72
Triglycerides (mmol/l)	<1.7	2.71	1.12	1.48	0.98	2.36
High density lipoprotein -cholesterol (mmol/l)	>1.0	1.12	1.24	1.07	1.31	1.27
Low density lipoprotein -cholesterol (mmol/l)	<3.0	2.19	3.06	3.4	2.82	2.38
Creatinine (μmol/L)	76–90	49	45	52	54	47
Creatine phosphokinase (U/L)	20–200	1,500	5,000	4,500	1,300	1,080
Thyroid stimulating hormone (mU/L)	0.4–4.0	1.3	1.3	12.86	1.5	0.4
Free thyroxine (pmol/L)	10.3–24.5	16.4	14.9	14.05	14.3	10.18
Glycated Hemoglobin (%)	4.0–6.0	4.9	5.5	4.9	4.7	5.3
Total testosterone level (ng/mL)	3.30–8.05	4.80	5.66	10.53	8.60	7.64
Neurofilament light (pg/mL)	<9.5^*^	10.56	4.37	5.20	10.01	20.62

* *Based on our control group data*.

NfL concentration was the highest for Patient 5 (20.62 pg/mL), followed by Patients 1 (10.56 pg/mL) and 4 (10.01 pg/mL). Our NfL control group consisted of 21 male participants, with a mean age of 27.3 ± 11.7 (range 5–55 years). There was no difference in age between the patient and control groups (*p* > 0.05). The mean NfL chain concentration among control group participants was 5.08 ± 1.76 pg/mL, ranging from 2.60 pg/mL to 8.80 pg/mL. Patient 5 had markedly increased NfL concentration in comparison with our control group, while Patients 1 and 4 also had higher NfL concentrations (>2.5 *SD*) than the control group. In total, SBMA patients group had higher NfL level than controls (*p* < 0.01).

## Discussion

In the current study, we have performed deep phenotyping and evaluation of possible biomarkers of all patients diagnosed with spinal and bulbar muscular atrophy in Latvia to unravel the extent of affected systems per patient, as well as to confirm that SBMA is a multisystemic condition.

Patients reported in this study exhibited typical features of SBMA with asymmetric atrophy and weakness of the proximal limb and bulbar muscles, as well as hand tremor consistent with lower motor neuron and skeletal muscle damage. Clinical, electrophysiological, and laboratory phenotyping of the patients allowed us to identify other symptoms and signs of SBMA that are rarely described or investigated. CAG repeat numbers in our SBMA group were variable (range 47–54). Although it has been reported that longer CAG repeats are associated with earlier onset of disease, our data did not show this association. However, our study group was very small. One reason for this could be that the onset of disease was reported by patients, while it could have started earlier or later than stated from the point of view of patients. CAG repeats are also associated with disease severity, which could have influenced our results. The mean CAG repeats in our group was 50.4, which was higher than previously reported (4).

Subclinical sensory involvement is common in patients with SBMA, affecting 70–100% of patients, even when motor nerve conduction studies are within reference range (3, 18). In SBMA, a typical feature is reduced sensory action potential amplitudes with axon loss, although decreased nerve conduction velocity and demyelination may also be present (19). In our study, all patients had small nerve fiber damage and two patients also had large nerve fiber damage detected in NCSs. Patient 1 had sensory demyelinating neuropathy, and Patient 5 had severe motor sensory demyelinating axonal polyneuropathy. There was no other confirmed cause for the severely manifested peripheral neuropathy. Sensory manifestations of SBMA in peripheral neuropathy and damage to both large and small nerve fibers are believed to be caused by the toxicity caused by accumulation of the mutant AR in dorsal root ganglion neurons in combination with metabolic alterations (18, 20, 21). Small-fiber involvement in SBMA could explain the neuropathic pain reported in some patients (22). Antonini et al. previously evaluated small nerve fiber involvement in a cohort of six patients with SBMA and were not able to find damage to small myelinated (A-δ) and unmyelinated (C types) nerve fibers by analyzing nerve biopsies of three patients with SBMA, although they found dysfunction in pain pathways among all six patients with SBMA that were investigated by using laser evoked potentials (23). However, another group was able to find the involvement of the small nerve fibers in two patients with SBMA by QST and skin biopsy analysis (20). Our data not only confirms the involvement of small nerve fibers in the pathogenesis of SBMA but also suggest that it may be a common feature among these patients.

The ANS is not considered to be affected during the course of SBMA, as patients usually do not exhibit symptoms suggesting ANS dysfunction. There is a study by Rocchi et al. analyzing ANS dysfunction in patients with SBMA, which analyzed five patients with SBMA without diabetes, impaired glucose tolerance, or cardiovascular diseases with five autonomic function tests (24). They observed subclinical dysfunction in the sympathetic and parasympathetic systems in four out of five patients (24). However, no patients exhibited symptoms of dysautonomia. Another group observed moderate autonomic skin denervation and reduced sweating in two patients with SBMA by analyzing skin biopsies and amount of sweating after pilocarpine stimulation (20). Our study confirms the involvement of the ANS and extends the knowledge of dysautonomic symptoms in patients with SBMA. Three out of five patients had involvement of the sympathetic or parasympathetic ANS, as shown by heart rate variability and sympathetic skin response test. It should be noted that two patients also demonstrated symptomatic ANS dysfunction. Patient 2 had tachycardia, and Patient 3 had tachycardia with increased sweating. Both patients were using beta-blockers to treat the tachycardia. Presentation of burning neuropathic pain in the distal extremities with dysautonomic symptoms, such as decreased sweating and difficulties with ejaculation, was recently reported in a patient with the highest number of CAG repeats known (68 CAG repeats) and confirms the involvement of small nerve fibers, as well as ANS, in the course of the disease (22). Previously reported damage to neurons from the AR toxicity in central neural autonomic regions (e.g., hypothalamus, nucleus ambiguous, and spinal intermediolateral nucleus, as well as in sympathetic ganglia) (21) could explain the involvement of the ANS among patients with SBMA. Additionally, it was suggested that ANS damage could also be caused by disruption of the small nerve fibers (post-ganglionic unmyelinated fibers) of the ANS (22). Altogether, our data, as well as previous data, show that the ANS is also involved in SBMAbut is rarely symptomatic. We find it peculiar that sensory polyneuropathy and ANS involvement were mutually exclusive. Polyneuropathy was reported in two patients, while ANS damage was reported in three patients. These findings are in agreement with the hypothesis that damage to different parts of the nervous system in SBMAmay have different pathogeneses.

Lower urinary tract symptoms (LUTS) without benign prostatic hyperplasia has been reported in up to 40% of the patients (25). The cause of LUTS in these patients is unknown and is hypothesized to be caused by androgen insensitivity. Despite the fact that none of our patients with dysautonomia reported LUTS, we hypothesize that at least some of the patients had LUTS as a result of ANS involvement (which was not recognized previously). It should be noted that LUTS could be present among our patient population but was not diagnosed, because it was not objectively evaluated using specific tools (e.g., International Prostate Symptom Score) in our patients.

Plasma NfL concentration is a known biomarker for various nervous system disorders in the central, as well as peripheral, nervous systems, reflecting axonal damage (26). A recent study investigated NfL as a biomarker of SBMA severity and found no difference in mean NfL levels between the SBMA patient and control groups, although several outliers with increased NfL levels were found (14). It should be noted that NfL level measurement was performed using the same method as in the previous study. In our study, three of five patients, however, demonstrated increased NfL levels. Two of these patients (Patients 1 and 5) had polyneuropathy confirmed by NCSs. Previous studies have shown that demyelinating and axonal forms of inherited neuropathies both have increased levels of NfL (27). We speculate that the NfL level for these two patients reflects peripheral nerve damage due to peripheral polyneuropathy. Patient 5 had severe polyneuropathy, as well as the highest NfL concentration. It has been reported that there is an age-dependent NfL increase, which could suggest that other factors were influencing the NfL level in Patient 5 (27). Patient 4 had increased NfL levels despite having no detectable polyneuropathy. Patient 4 had a BMI at the lower threshold of normal values (18.7 kg/m^2^), which has been reported to result in a NfL increase in patients with anorexia nervosa (28). We hypothesize that increased NfL reflects the involvement of additional systems (peripheral nervous system) or other factors (age, BMI and other). The previous study by Lombardi et al. (14), which did not find NfL level differences between patients with SBMA and controls, did not describe whether patients with SBMA had involvement of additional systems. Therefore, we hypothesize that NfL can be used as a biomarker of peripheral polyneuropathy in SBMA but requires validation in a larger cohort with detailed evaluation of multisystemic involvement and other influencing factors, possibly in patients with SBMA from our geographic region.

All patients in our study had increased creatine phosphokinase levels, indicating muscle damage, and normal creatinine levels. Elevation of creatine phosphokinase in patients with SBMA could reflect myopathic damage. Creatinine for all our patient cohort was under reference range reflecting possible muscle damage (29). Laboratory studies confirmed a high prevalence of dyslipidaemia (reported in 4/5 patients) and a low prevalence of diabetes (no patient in our study group), as assessed by the glycated hemoglobin, which was similar to other studies (2, 18, 30) although impaired glucose tolerance, which is common, was not assessed. Endocrinopathies (thyroid disorders or partial androgen insensitivity) is also common and affected all our patients. The metabolic abnormalities are believed to be caused by androgen insensitivity.

Heart muscle is usually spared despite the well-known primary myopathic damage in SBMA and AR accumulation that occurs in the cardiomyocyte nuclei (18, 31). Araki et al. described the common occurrence of Brugada syndrome in up 12% of patients with SBMA from Japan (31). Occurrence of Brugada syndrome was also observed among Caucasian patients with SBMA, although at a much lower frequency (up to 4%) (25). We were not able to find electrocardiographic abnormalities (excluding tachycardia in Patients 2 and 3), which was most probably due to the low frequency of cardiac involvement.

The main limitation of this study is the small sample size. However, we were able to analyse most of the symptoms reported in patients with SBMA, as well as replicate and confirm previous findings, to provide the information about the extent of affected systems by deeply phenotyping five patients with SBMA. Other features and symptoms reported in SBMA patients that were not analyzed in our study were only a subclinical change in brain metabolism, osteopenia, and non-alcoholic steatohepatitis (18, 32, 33). Additionally, we were able to raise hypotheses that should be validated in larger cohorts. Besides the limited number of patients, another bias of our study is that our patients are younger than those analyzed in other studies, and the mean CAG repeat count was higher than in other studies. Unfortunately, muscle, skin or nerve biopsy was not available for further examination.

Our study confirms the systemic involvement in patients suffering from SBMA and expands knowledge about some of the features regarding ANS and small nerve fiber involvement, which have been inconclusive in previous studies. Our data show that small nerve fibers and the ANS are commonly affected in patients with SBMA. In our study, NfL showed biomarker capabilities with regard to the peripheral polyneuropathy in SBMA but requires validation in a bigger well-phenotyped cohort, where more factors influencing the NfL levels in patients are known. The complex phenotype of SBMA should be kept in mind, as it could help to identify and diagnose patients with SBMA, as well as provide explanations for the non-typical symptoms patients may have. In the age of therapy of genetic disorders, correct and timely diagnosis could also help in providing timely treatment for patients.

## Data Availability Statement

The original contributions presented in the study are included in the article/supplementary material, further inquiries can be directed to the corresponding author/s.

## Ethics Statement

The studies involving human participants were reviewed and approved by the Central Medical Ethics Committee of the Republic of Latvia (Nr.3/18-03-21). The patients/participants provided their written informed consent to participate in this study. Written informed consent was obtained from the individual(s) for the publication of any potentially identifiable images or data included in this article.

## Author Contributions

VK, HZ, LG, and KB were involved in planning and supervised the study. EM, IG, and VK performed the clinical evaluation. DR, LG, and GT performed genetical analysis. EM, DR, and IG drafted the manuscript. NK, VP, VK, LG, GT, HZ, and KB made contribution to revising the paper and interpreting the results. All authors read and provided critical feedback on manuscript draft as well as approved the final version of manuscript.

## Conflict of Interest

VP was employed by BIOCON Medical Laboratory, LCC BIOCON. HZ has served at scientific advisory boards for Denali, Roche Diagnostics, Wave, Samumed, Siemens Healthineers, Pinteon Therapeutics, and CogRx, has given lectures in symposia sponsored by Fujirebio, Alzecure, and Biogen, and is a co-founder of Brain Biomarker Solutions in Gothenburg AB (BBS), which is a part of the GU Ventures Incubator Program (outside submitted work). The remaining authors declare that the research was conducted in the absence of any commercial or financial relationships that could be construed as a potential conflict of interest.
